# Prevalence and Characterization of Methicillin-Resistant *Staphylococcus aureus* from Animals, Retail Meats and Market Shopping Vehicles in Shandong, China

**DOI:** 10.3390/foods15020248

**Published:** 2026-01-09

**Authors:** Ting-Yu Yang, Chong-Xiang Sun, Junjie Wang, Zhiyuan You, Hao Wang, Kelan Yi, Feng-Jing Song, Bao-Tao Liu

**Affiliations:** 1 College of Veterinary Medicine, Qingdao Agricultural University, Qingdao 266109, China; younglucky9907@163.com (T.-Y.Y.); 16634254313@163.com (J.W.); youzhiyuan6029@163.com (Z.Y.); 15215488657@163.com (H.W.); 18372375108@163.com (K.Y.); 2 Weifang Animal Disease Prevention and Control Center, Weifang 261000, China; scx6012@163.com; 3 Institute of Plant Protection, Qingdao Academy of Agricultural Sciences, Qingdao 266100, China

**Keywords:** MRSA, retail meats, shared shopping vehicles, enterotoxin genes

## Abstract

*Staphylococcus aureus* has been recognized as an important foodborne pathogen and methicillin-resistant *S. aureus* (MRSA) can cause fatal infections worldwide. Of great concern is that MRSA have been found in animals and non-healthcare settings; however, knowledge about the prevalence and genetic characteristics of *S. aureus*, especially MRSA from animals, retail meats and market shared shopping vehicles in the same district, is limited. In this study, we collected 423 samples including handrail swabs (*n* = 226) of shopping trolleys and baskets from 18 supermarkets, retail meats (*n* = 137) and swine nasal swabs (*n* = 60) between 2018 and 2020 in China. *S. aureus* isolates were isolated and identified by PCR, and then the *mecA* was used to confirm the MRSA. The antibiotic resistance and virulence genes among *S. aureus* were also analyzed, followed by whole genome sequencing (WGS). *S. aureus* isolates were widely distributed in shared shopping vehicles (8.0%, 18/226), retail meats (14.6%, 20/137) and swine (18.3%, 11/60). In total, 49 *S. aureus* were obtained and 20 of the 49 isolates were MRSA. We firstly reported a high prevalence of MRSA in shared shopping vehicles (7.5%, 17/226), followed by raw meats (2.2%, 3/137), and 44.4% (8/18) of the 18 supermarkets possessed MRSA-positive shopping vehicles. All 20 MRSA isolates were SCC*mec* IVa MRSA clones. Enterotoxin genes (*sea*/*seb*) associated with *S. aureus* food poisoning were present in 45.0% of the 20 *S. aureus* isolates from retail meats and 25.0% of the 20 MRSA isolates carried enterotoxin genes. Retail meats in this study carried ST6-MRSA, a common ST type of *S. aureus* from food-poisoning outbreaks in China. WGS showed that the MRSA from meats harbored enterotoxin gene *sea* and immune evasion genes (*sak* and *scn*) associated with human infections, and were clustered with previously reported MRSA isolates from animals and humans. The MRSA isolates carrying multiple virulence genes from shopping vehicles were also clustered with previously reported MRSA isolates from humans and animals, suggesting that the exchange of MRSA isolates might occur among different niches. Our results highlighted the risk of retail meats and shared shopping vehicles in spreading antimicrobial-resistant pathogens including MRSA. To our knowledge, this is the first report of the wide spread of MRSA in shared shopping vehicles in China.

## 1. Introduction

Antimicrobial resistance (AMR) is a critical One Health issue that has been linked to humans, animals and environments [1,2], and it often poses great threat to the health of both humans and animals [3]. *Staphylococcus aureus* (*S. aureus*) is a human pathogen and the presence of resistance genes exacerbates the risk of spreading antimicrobial resistances. *S. aureus* caused over 100,000 cases of bacteremia in the United States in 2017 [4]. In the European Union, it caused 434 foodborne outbreaks in 2015 and the estimated cost of a single outbreak of foodborne illness in a restaurant might exceed 1 million US dollars [5]. In China, *S. aureus* was one of the leading pathogens, responsible for causing 577 outbreaks, 9092 cases of illness and 3715 hospitalizations from 2010 to 2020 [6]. Methicillin-resistant *Staphylococcus aureus* (MRSA) can cause fatal infections worldwide [7], and 31.4% of all clinical isolates have been recognized as MRSA according to the China Antimicrobial Surveillance Network (CHINET) surveillance system in 2019 [8]. In most Asian countries, MRSA accounted for more than one-half of the hospital-related infections caused by *S. aureus* [9].

Besides those hospital-acquired MRSA (HA-MRSA) initially confined to hospitals, swine-derived ST398-type MRSA known as livestock-associated MRSA (LA-MRSA) was firstly reported to cause human infection in The Netherlands in 2005 [10]. The presence of MRSA strains in swine has been detected in several regions of China [11,12] and it is found that swine are important hosts for LA-MRSA. MRSA has also been isolated from cattle, horses, goats, rabbits, poultry, cats, dogs and food of animal origin [13,14,15,16], although the prevalence of MRSA in animals is low. Of particular concern is the fact that LA-MRSA carries AMR genes and can be transmitted between animals and humans [17]. The LA-MRSA may even cause human infections through direct or indirect contact with infected animals, posing a challenge to human health [18,19]. The presence of MRSA in the human food chain, especially in raw meats, has been reported [20,21]; however, the prevalence of MRSA varied between meats of different origins (such as beef, poultry meat and pork) and countries.

MRSA in food animals may not only cause animal diseases and contaminated food [22,23,24], but also contaminate farm environments, which are important vectors mediating the transmission of AMR or pathogens between animals and humans. Of great concern is that MRSA have been isolated and characterized in several non-farm environments such as public shared bicycles [25,26] and metro system [27]. The high volume of people in public areas and the survival of MRSA on public surfaces facilitate the transmission of Staphylococci including MRSA in such areas. Supermarket shopping is still the most common shopping form, and shared shopping vehicles in supermarkets have been an indispensable component of people’s daily life. Despite great convenience, the shared shopping vehicles could be a source of antimicrobial-resistant pathogenic bacteria, posing threat to public health. However, there remains a paucity of data regarding the distribution and features of MRSA from shopping vehicles in supermarkets. The association of *S. aureus*, especially MRSA from retail meats, shopping vehicles and animals, should be investigated, and the role of shopping vehicles in disseminating pathogens, especially MRSA, remains largely unknown.

China has huge amounts of supermarkets, consumers of raw meats and food animals, creating an ideal situation for the spread of *S. aureus* including MRSA. In this cross-sectional study, we aimed to elucidate the prevalence and molecular characteristics of *S. aureus* strains, particularly MRSA, among shopping vehicles within supermarkets, raw meats and swine in Shandong Province of China. The association of the MRSA isolates from different niches was also analyzed.

## 2. Materials and Methods

### 2.1. Sample Collection and Identification of Strains

A total of 423 samples from 5 cities (Qingdao, Zibo, Heze, Weifang, Dezhou) of Shandong province in China were collected between 2018 and 2020. These samples included swine nasal swabs (*n* = 60), raw meats (*n* = 137) and shared shopping vehicles within supermarkets (*n* = 226) (Table 1).

Nasal swabs were collected from 60 healthy pigs which were randomly selected from 2 pig farms in city Qingdao, China. Chicken meat (*n* = 94) and pork (*n* = 43) samples were purchased from nine supermarkets in city Qingdao. Each raw meat sample was aseptically homogenized and 25 g of homogenized sample was mixed with 225 mL Luria-Bertani (LB) broth (Hopebio, Qingdao, China) containing 7.5% sodium chloride and incubated at 37 °C for 16 to 24 h. The handrails of 170 shopping trolleys and the handles of 56 shopping baskets from 18 supermarkets in 5 cities were wiped with sterile cotton swabs soaked with saline. Of the 18 supermarkets, 6 were from those we collected meat samples in city Qingdao and 78 samples (60 from shopping trolleys and 18 from baskets) were collected. Three supermarkets were selected from each of the other four cities and there were 40, 39, 40 and 29 samples collected from city Heze, Zibo, Dezhou and Weifang, respectively (Table 2). Each swab was placed into sterile LB broth containing 7.5% NaCl and these samples were stored below 8 °C and processed within 24 h after sampling.

The LB broth with survived bacteria was diluted in series of 1:10 and 100 µL of appropriate dilution and was spread onto CHROMagar^TM^ Staph aureus medium (CHROMagar, Paris, France) and incubated for 24 h. The colonies with pink were suspicious *S. aureus* and one colony showing typical appearance was selected from each sample. DNA extraction was performed using the phenol–chloroform method as previously described, with minor modification [28]. Briefly, the bacteria in 325 μL TE buffer (10 mM Tris, 5 mM EDTA, pH 7.8) was cultured with 25 μL lysozyme solution (10 mg/mL) at 37 °C for 1 h. Lysis of cells was achieved by addition of 20 μL of SDS (20% SDS *w*/*v* in Tris (50 mM), EDTA (20 mM), pH 7.8) followed by adding 3 μL of proteinase K (20 mg/mL, Sigma, St. Louis, MO, USA) and further incubated at 37 °C for 1 h. Protein was precipitated by the addition of 200 μL NaCl (5 M) followed by centrifugation (8000 rpm) for 10 min. The supernatant was extracted with phenol/chloroform (1:1) followed by chloroform/isoamyl-alcohol (24:1) extraction. DNA was precipitated from the supernatant by adding 0.6 volumes of isopropanol and collected by centrifugation at 8000 rpm, 4 °C for 10 min. The pellet was washed with 70% ethanol and dried, followed by being resuspended in 1 mL of TE buffer. Identification of *S. aureus* was performed by using the *nuc* and *femB* genes as previously described [29,30].

### 2.2. Antimicrobial Susceptibility Testing

The antimicrobial susceptibilities of the obtained 49 *S. aureus* isolates to 11 antimicrobials were determined using the agar dilution method according to the guidelines of Clinical and Laboratory Standards Institute (CLSI) [31]. The 11 antimicrobial agents were as follows: penicillin, ciprofloxacin, levofloxacin, vancomycin, linezolid, gentamicin, kanamycin, clindamycin, erythromycin, tetracycline and florfenicol. The breakpoints for these antimicrobials were recommended by the CLSI [31]. The reference strain *S. aureus* ATCC 25923 was used for quality control.

### 2.3. Detection of mecA, Virulence Genes and Antibiotic Resistance Genes

All *S. aureus* isolates were further screened for the presence of *mecA* to confirm the MRSA [32]. Thirteen virulence genes encoding the Panton–Valentine leukocidin (*lukF-PV/lukS-PV*), enterotoxins (*sea*, *seb*, *sec*, *sed*, *see*), toxic shock syndrome toxin-1 (*tsst-1*), exfoliative toxins (*eta*, *etb*), hemolysin (*hla*, *hlb*) and the adhesion factors (*fnbA*, *fnbB*) were screened using PCR as described previously [33,34,35,36]. The common macrolide resistance genes *erm(A)*, *erm(B)* and *erm(C)* among all *S. aureus* isolates were analyzed by PCR as previous described [37]. The presence of vancomycin resistance genes *vanA* and *vanB* were further investigated according to the previous method [38]. The oxazolidinones resistance genes *cfr*, *poxtA* and *optrA* were also screened as described previously [39]. The primers used in this study were listed in Table S1.

### 2.4. SCCmec Typing

SCC*mec* types and subtypes I, II, III, IVa, IVb, IVc, IVd and V of the MRSA isolates in this study were identified by multiplex-PCR using specific primers (Table S1), as previously described [40]. PCR conditions were set at 94 °C for 5 min, followed by 30 cycles of 94 °C for 30 s, 52 °C for 45 s and 72 °C for 90 s, ending with a final extension step at 72 °C for 10 min. The PCR amplicons were electrophoretically separated at 100 V/cm in a 1.5% agarose gel. MRSA isolates that could not be assigned to type I–V were defined as nontypeable (NT).

### 2.5. Whole-Genome Sequencing

All three MRSA isolates from retail meats and two randomly selected MRSA isolates from shopping vehicles in Qingdao were subjected to whole-genome sequencing (WGS). The methicillin-sensitive *S. aureus* (MSSA) isolate HD15L3 from shopping trolley in Qingdao and one ciprofloxacin-resistant MSSA isolate 2L5 from pig in Qingdao were also subjected to WGS. Briefly, total DNA of the seven *S. aureus* isolates was extracted using a TIAN amp Bacteria DNA kit (Tiangen, Beijing, China) and then sequenced using an Illumina HiSeq 2500 platform (Illumina, San Diego, CA, USA) with the 150 paired-end sequencing strategy. Illumina reads were quality checked using software FastQC version 0.11.9 (https://www.bioinformatics.babraham.ac.uk/projects/fastqc/) (accessed on 10 June 2022). SPAdes v 3.12.0 was used to assemble the clean Illumina reads [41]. The assembled sequences were subjected to CGE (http://www.genomicepidemiology.org/services/) (accessed on 2 June 2024) to analyze the antibiotic resistance genes. Virulence genes were also analyzed using CGE (https://www.genomicepidemiology.org/services/) (accessed on 2 June 2024) and VFDB core dataset (http://www.mgc.ac.cn/VFs/main.htm) (accessed on 3 June 2024). Functional annotation was performed using the NCBI Prokaryotic Genome Annotation Pipeline server.

To track the phylogenomic relationships of the *S. aureus* isolates in our study and those reported from food, humans and animals, we used the seven genomes in this study and the assembled genomic sequences of 70 *S. aureus* isolates in the NCBI database to obtain the phylogenomic tree. The 77 genomes consisting of 56 MRSA isolates and 21 MSSA isolates included 12 genomes from animals, 51 from humans, 5 from environment and 9 from food (Table S2). SNPs were determined as we described previously [42]. The MRSA isolate HD33L3 from shopping trolley was set as reference. The phylogenetic tree was visualized using Evolview version 3 [43].

### 2.6. Statistical Analysis

Comparisons between genotype and antibiotic resistance frequencies in different populations (sample types, MRSA/MSSA) were performed using the Pearson Chi-square test in SPSS software (version 21). False discovery rate according to Benjamini–Hochberg procedure was applied to adjust for multiple comparisons. All tests of significance were two-tailed, and a *p*-value of ≤0.05 was considered to be statistically significant.

## 3. Results

### 3.1. Prevalence of S. aureus and MRSA

A total of 49 isolates were positive for both the *nuc* and *femB* genes and were classified as *S. aureus*. The overall prevalence of *S. aureus* among the 423 samples was 11.6% (Table 1). *S. aureus* was found in 18.3% of the swine samples, which was similar to that (14.6%, 20/137) in retail raw meats. The detection rate of *S. aureus* in chicken meats and pork was 17.0% (16/94) and 9.3% (4/43), respectively. Among the 226 samples from shopping vehicles, 18 (8.0%) harbored *S. aureus* and there was no significant difference between the isolation rate from shopping trolleys (7.6%, 13/170) and that from shopping baskets (8.9%, 5/56) (*p* > 0.05) (Table 1). MRSA isolates were identified in 20 samples in this study. Notably, 17 of the 226 (7.5%) shopping vehicles samples carried MRSA and the detection rate of MRSA in shopping trolleys and baskets was 7.1% and 8.9%, respectively. There were only three chicken meat samples that harbored MRSA, while no MRSA was found in pork and swine nasal swabs (Table 1).

Shopping vehicles with MRSA were found in all five cities (Table 2). Of the five cities, the highest detection rate of shopping vehicles carrying MRSA was found in Heze (17.5%, 7/40), followed by Weifang (13.8%, 4/29) (Table 2). Two of the three supermarkets in city Heze, Dezhou and Weifang harbored MRSA-positive samples. In total, 8 of the 18 supermarkets (44.4%) in this study possessed MRSA-positive shopping vehicle samples. Notably, the detection rates of MRSA in five supermarkets were above 14.3% and 28.6% of the shopping vehicles from market 9 in Heze carried MRSA (Table 2).

### 3.2. Antimicrobial Susceptibility and Antibiotic Resistance Genes

As shown in Table 3, 89.8% of the 49 *S. aureus* isolates were resistant to erythromycin, followed by clindamycin and penicillin. Resistance rates to ciprofloxacin, tetracycline, kanamycin and florfenicol were all above 30.0%. Notably, the resistance rate to ciprofloxacin and florfenicol among *S. aureus* isolates from animals was significantly higher than that from raw meats and that from shopping vehicles, respectively (*p* < 0.05) (Table 3). The resistance rate to kanamycin among *S. aureus* isolates from shopping vehicles was significantly higher than that from raw meats (*p* < 0.05) (Table 3). Among MSSA isolates, the resistance rate to ciprofloxacin, tetracycline, gentamicin, levofloxacin and florfenicol was significantly higher than that of MRSA isolates, respectively (*p* < 0.05) (Table 3).

Among the 49 *S. aureus* isolates, 44.9% and 40.8% carried macrolide resistance genes *erm(B)* and *erm(C)*, respectively (Table 4). Resistance genes *erm(A)*, *vanA*, *vanB*, *cfr*, *poxtA* and *optrA* were not found in this study. The detection rate of *erm(B)* among shopping vehicles *S. aureus* isolates was significantly higher than that from raw meats (*p* < 0.05), while the detection rate of *erm(C)* in *S. aureus* isolates among animals, raw meats and shopping vehicles was not significantly different (*p* > 0.05). Both the detection rate of *erm(B)* and *erm(C)* among MRSA isolates were not significantly different from MSSA isolates (*p* > 0.05).

### 3.3. Virulence Genes in S. aureus Isolates from Different Origins

Seven of the thirteen screened virulence genes were found in this study. Among the 49 isolates, 100.0%, 85.7% and 65.3% carried *hla*, *hlb* and *fnbB*, respectively, followed by *eta* (46.9%), *sea* (14.3%), *seb* (10.2%) and *lukF-PV/lukS-PV* (6.1%) (Table 4). The prevalence of enterotoxin genes (*sea/seb*) associated with food poisoning in *S. aureus* isolates was 22.4% (11/49) and enterotoxin genes were present in 45.0% of the 20 *S. aureus* isolates from retail meats. Five of the 20 MRSA (25.0%) carried enterotoxin genes. The detection rate of *eta* in *S. aureus* isolates from raw meats was significantly higher than that from shopping vehicles and that from animals (*p* < 0.05). The detection rate of *sea* among *S. aureus* isolates from raw meats was significantly higher than that from animals (*p* < 0.05). The detection rate of *lukF-PV/lukS-PV* and *sea* in MRSA isolates was significantly higher than that in MSSA isolates, respectively, while the detection rate of *eta* and *fnbB* in MSSA isolates was significantly higher than that in MRSA isolates, respectively (*p* < 0.05).

### 3.4. SCCmec Typing of MRSA Isolates and Whole-Genome Sequence Analysis

All the 20 MRSA isolates belonged to the SCC*mec* type IVa. WGS analysis showed that all three MRSA isolates (105L1, 105L2 and 308) from raw meats belonged to ST6 and possessed not only the *mecA* but also *bla*_Z_, *erm(C)* and *grlA*: S80F (Table 5). Unlike the three MRSA isolates from raw meats, both MRSA isolates (HD33L3, HD36L3) from shopping vehicles carried *mecA*, *erm(B)*, *aph(3′)-III* and *ant(6)-Ia.* Compared to ST59 HD33L3, the ST338 type HD36L3 carried two additional resistance genes including *tet(K)* and *bla*_Z_. Both MSSA isolates (2L5, HD15L3) belonged to ST398 and carried 14 resistance genes encoding resistances to aminoglycosides, fluoroquinolones, tetracyclines, folate pathway antagonist, beta-lactams, amphenicols, macrolides, pleuromutilin and lincosamide (Table 5). An additional *tet(K)* (tetracycline resistance) was also found in isolate HD15L3.

All five sequenced MRSA isolates from raw meats and shopping vehicles harbored six to seven classes of virulence genes, including genes related to adherence, exoenzyme, exotoxin, effector delivery system, immune modulation, nutritional/metabolic factor and biofilm factor (Table 5). Only MRSA isolate 105L2 from raw meat carried all seven classes of virulence genes. Adherence gene (*eap*/*map*) was only found in the three MRSA isolates from raw meats, among which two lacked virulence genes for biofilm factor. Biofilm factors were found in five sequenced *S. aureus* isolates, including the three from shopping vehicles. Only MRSA isolate HD36L3 recovered from shopping baskets had the Panton-Valentine leucocidin toxin gene *lukS-PV* and the *Staphylococcus* enterotoxin B gene *seb*. All three MRSA from retail meats harbored the immune evasion cluster (IEC) with the *sea* and *sak* genes. Overall, 35 (*n* = 2), 32 (*n* = 2), 45 (*n* =1), 40 (*n* =1), and 33 (*n* = 1) virulence genes were detected in the seven WGS-sequenced *S. aureus* isolates.

**Figure 1:**
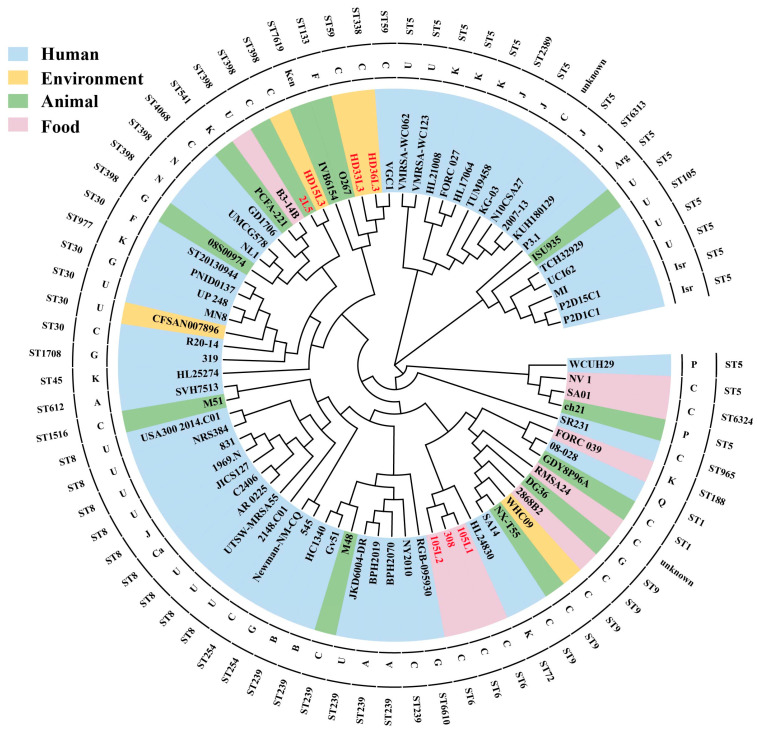
Phylogenetic analysis of the 77 *S. aureus* genomes from different countries and different sources, including 70 genomes from the NCBI database and 7 isolates in this study. U, USA; J, Japan; K, Korea; B, Brazil; C, China; F, France; G, Germany; P, Poland; A, Australia; N, Netherlands; Arg, Argentina; Isr, Israel; Ken, Kenya. Ca, Canada. The seven genomes marked with red font were from this study.

A total of 23, 170 SNPs were obtained from the 77 *S. aureus* genomes of different sources and *S. aureus* isolates belonging to the same STs were clustered closely (Figure 1). All three ST6 MRSA isolates from raw meats in our study were clustered together and had a limited number of variations (362 SNPs), indicating that a close genetic relationship existed among these isolates, even for those from different samples or supermarkets. Notably, our three MRSA isolates from raw meats were also clustered closely with MRSA isolate HL24830 (CP082789) from human blood in South Korea. The MRSA isolate NX-T55 (CP031839) from animal in China showed less than 192 SNPs away from the MRSA 2868B2 (CP060141) in food, MRSA isolate WHC09 (CP077755) from animal farm bioaerosol and MRSA isolate SA14 (CP073012) from human in China. These results suggest that the exchange of MRSA isolates might occur among different niches and the MRSA in retail meats will pose a threat to public health.

Furthermore, both the MRSA isolates HD33L3 and HD36L3 from shopping vehicles in Qingdao supermarket were clustered with MRSA isolate VG1 (CP039448) from human blood and O267 (CP034102) from animal. The ST398 isolate 2L5 from animal in this study was closely related to the ST398 isolate HD15L3 from shopping trolley, which was also clustered with the animal ST398 MRSA isolate 08S00974 (CP020019) and human ST398 MRSA isolates NL1 (CP077741) and UMCG578 (CP077738). These results further suggest that exchange of MRSA isolates might occur among different niches, especially between shopping vehicles and humans.

## 4. Discussion

The contamination of animals and retail meats by *S. aureus*, especially MRSA isolates expressing virulence genes and resistance genes, remains an urgent public health issue [19]. The MRSA isolates from animals can spread to animal meats and are also a significant source of human MRSA by direct or indirect contact [44]. The prevalence of *S. aureus* in animals in this study (18.3%) was higher than that in animals (5.4%) from Xinjiang, China [45] and that (10.1%) in pigs in Chongqing, China [46], but significantly lower than that (65.4%) in other commercial farms from Shandong, China [47]. Unlike those previous studies [47,48,49,50], no samples from the two swine farms in this study carried ST398 MRSA, which might be due to the very low MRSA prevalence in the randomly selected farms in our study. Only one strain from one sample in this study might also inevitably miss some drug-resistant bacteria, including MRSA. *S. aureus* was identified in 14.6% of raw meat samples in our study, significantly lower than that (69.0%) in Danish retail meats [24] and that (33.6%) in Cambodian retail meat [51]. The prevalence of MRSA in retail meats in the current study was 2.2%, which was similar to that from Xinjiang (2.0%) China [45], but significantly lower than the detection rate of MRSA (13.1%) in retail meats in Denmark [24].

MRSA have also been detected in several public transportation systems such as public shared bicycles [25,26]. We presented that *S. aureus* isolates were widely distributed among shared shopping vehicles from supermarkets in Shandong, China. Of particular concern, we reported for the first time that MRSA was observed in shared shopping vehicles. Notably, eight of the eighteen supermarkets (44.4%) in this study possessed MRSA-positive shopping vehicle samples, which were distributed in all five cities studied, suggesting that MRSA isolates might have been prevalent in shopping vehicles in China. The overall high prevalence of MRSA (7.5%) in shopping vehicles in this study was higher than that in the Guangzhou metros in China (2.5%) [27], general public settings in London, UK (0.0%) [52] and shared bicycles in Chengdu, China (1.0%) [26], however, lower than that among public buses in the USA (62.5%) [53]. The high prevalence of MRSA in handrails of shopping vehicles in this study suggests that humans have contaminated the shopping vehicles, which will pose a threat to humans.

Virulence factors in *S. aureus* play an important role in pathogenesis [54]. A recent survey in China reported that only 3.9% MRSA strains harbored the *tst-1* gene [55], and similarly, we did not find *tst-1* in our study. Notably, the frequency of *eta* in *S. aureus* from human in Hainan, China was 57.3% [28] and 46.9% of the *S. aureus* in our study carried *eta*, much higher than those from human in central-southern China [56]. The Panton–Valentine leukocidin genes *lukF-PV/lukS-PV* has been proved to be highly pathogenic [57], and community-acquired *lukF-PV/lukS-PV*-associated necrotizing pneumonia infecting previously healthy people is often fatal [58]. The *lukF-PV/lukS-PV*-positive *S. aureus* isolates are more likely to cause skin and soft tissue abscesses and deep abscesses than *lukF-PV/lukS-PV*-negative isolates [58]. Among the MRSA isolates in this study, 15.0% carried the *lukF-PV/lukS-PV* gene and all *lukF-PV/lukS-PV*-positive MRSA isolates were from shared shopping vehicles, significantly higher than a previously study in which none of the MRSA isolates in metro system carried *lukF-PV/lukS-PV* [27]. However, the prevalence of *lukF-PV/lukS-PV* in *S. aureus* isolates in this study (6.1%) was lower than that in *S. aureus* isolates from humans in Hainan, China (47.6%) [28]. The enterotoxins are the main virulence factors that can trigger food poisoning and foodborne illness, causing fever and shock, even after the bacteria have been destroyed by heating [59]. Five classical enterotoxins, including *sea*, *seb*, *sec*, *sed* and *see*, have been considered to be associated with *S. aureus* food poisoning [60], and enterotoxin genes (*sea*, *seb*) were present in 45.0% of the *S. aureus* isolates from retail meats in this study, which will pose a threat to food safety. Of note, three MRSA isolates from retail meats in this study harbored the immune evasion cluster (IEC) system with the *sea* and *sak* genes, which is a specific marker of adaptation to the human host [61]. The prevalence of IEC in the 20 MRSA isolates (20.0%) was higher than that (2.8%) in MRSA isolates from retail meats in the Czech Republic [20]. In addition, MRSA isolate HD36L3 recovered from shopping basket had both the Panton–Valentine leucocidin toxin gene *lukS-PV* and *seb*, one of the five classical enterotoxins associated with food poisoning [60], suggesting that MRSA isolates in shared shopping vehicles will pose a threat to humans.

The resistance rate to ciprofloxacin and florfenicol among animal-sourced *S. aureus* isolates was significantly higher than that from raw meats and that from shopping vehicles, which might be related to the massive use of antibiotics in pig farms. MRSA isolates resistant to several antibiotics often result in the failure of treating infections. In this study, the MSSA isolates had higher resistance to ciprofloxacin, tetracycline and florfenicol than that in MRSA. This might be because the MSSA were mostly isolated from raw meats and animals, which were often treated with antibiotics, while MRSA were mostly from shared shopping vehicles in supermarkets. Most of the MSSA isolates might carry resistance genes, accounting for the resistances to antibiotics mentioned above, as shown in the WGS results, leading to the higher antibiotic resistances among MSSA isolates in this study. With WGS analysis, we found six resistance genes, conferring resistance to ≥3 classes of antibiotics including macrolides, in the five WGS-sequenced MRSA isolates from retail meats and shared shopping vehicles. The macrolide resistance genes *erm(B)* and *erm(C)* were not only prevalent in *S. aureus* isolates but also in MRSA isolates especially those from shared shopping vehicles. These results highlight the important role of shared shopping vehicles in maintaining a reservoir of antimicrobial resistance. Antimicrobial-resistant bacteria and resistance genes are constantly enriched in the environment and spread through the environment to humans via horizontal transfer [62,63].

The three MRSA isolates from retail meats in this study were ST6, a common ST type of *S. aureus* from food-poisoning outbreaks [64,65] and shared bicycles [26] in China. ST59, a common ST type of MRSA clone in both humans [66] and shared bicycles [26] in China, was also found in shared shopping baskets in this study. ST398-MSSA was a prevalent ST type in humans in China [66] and ST398-MSSA carrying 14~15 resistance genes was also present in animal and shopping trolley in the current study. Contact with animals infected by ST398 *S. aureus* has been considered one of the major risk factors for human infection. All MRSA isolates from retail meats and shopping vehicles in this study belonged to the SCC*mec* type IVa, which was also the most prevalent MRSA clone in hospitals in Hainan, China [28], indicating a correlation between MRSA isolates from retail meats, environment and humans. Our results suggest that both retail meats and shared shopping vehicles are important reservoirs of pathogens.

Based on WGS analysis, the MRSA isolates from retail meats were clustered closely with MRSA isolates from animals and humans in China, suggesting that the MRSA isolates in food might be derived from animals and could be transmitted to humans via the food chain. The ST398 isolate from animal in this study was closely related to the previously reported animal ST398 MRSA isolates and human ST398 MRSA isolates, supporting the possibility of ST398 MRSA transmission between animals and humans. The animal and human ST398 MRSA isolates were also clustered with the ST398 isolate from shopping trolley in this study, indicating that shared shopping vehicles are likely to play an important role in bacterial transmission. Importantly, the MRSA isolates from shopping vehicles in this study were clustered with MRSA isolates from human blood and animals. These results confirm that exchange of MRSA isolates occurred among different niches. Shopping vehicles might be contaminated from human, either from human infection or humans touching contaminated food/animals, and shopping vehicles in supermarkets may participate in transmitting MRSA isolates among humans. Combination of hand hygiene and disinfection of shopping vehicles handrails before and after use, is recommended to prevent MRSA transmission.

We acknowledge that this study has some limitations. First, the sample size was geographically limited and may not reflect the prevalence of MRSA especially in animals and retail meats from Shandong, China. Second, only a small number of isolates were selected for WGS in this study and the genomes of the remaining MRSA have not yet been analyzed, which might limit the phylogenetic inferences. The genomic information on a wide range of isolates will provide a more detailed genotypic characteristics of MRSA. Additionally, the corresponding humans have not been sampled because the humans consuming the retail meats or touching the shopping vehicles in our study are difficult to trace. Future research should include broader geographic sampling and more genome data to jointly decipher the molecular exchange mechanisms of MRSA isolates among different niches.

## 5. Conclusions

In summary, *S. aureus* isolates are widely distributed in animals, retail meats and shared shopping vehicles in Shandong province of China. We report for the first time a high prevalence of MRSA (7.5%) in shared shopping vehicles, followed by retail meats (2.2%), suggesting that both retail meats and shared shopping vehicles serve as an important reservoir of MRSA. The MRSA isolates from retail meats/shared shopping vehicles in this study were clustered with previously reported MRSA isolates from animals and humans in China, confirming that exchange of MRSA isolates occurred among different niches, which further revealed that the MRSA isolates in food and shared shopping vehicles might pose a threat to humans. Nationwide epidemiology studies about MRSA are needed to fully assess the risk of retail meats and shared shopping vehicles in spreading diseases. To our knowledge, we first reported the widespread of MRSA in shared shopping trolleys and baskets in China.

## Figures and Tables

**Table 1:** Isolation rates of *S. aureus* and MRSA in different types of samples.

Sources	Sample Types	Number of Samples	Isolation Rates of *S. aureus* (%)	Isolation Rates of MRSA (%)
Swine farms	Nasal swabs	60	18.3 (11/60)	0 (0/60)
Raw meats			14.6 (20/137)	2.2 (3/137)
	Pork	43	9.3 (4/43)	0 (0/43)
	Chicken meats	94	17.0 (16/94)	3.2 (3/94)
Shopping vehicles			8.0 (18/226)	7.5 (17/226)
	Shopping trolleys	170	7.6 (13/170)	7.1 (12/170)
	Shopping baskets	56	8.9 (5/56)	8.9 (5/56)
Total		423	11.6 (49/423)	4.7 (20/423)

**Table 2:** Isolation rates of *S. aureus* and MRSA from shopping vehicles in different supermarkets and cities.

Area	Supermarkets	Sample Type	Number of *S. aureus*-Positive Samples	MRSA Rate in Samples of Each Supermarket (%)	MRSA Rate in Samples of Each City (%)
Qingdao (*n* = 78)	Market 1	Shopping trolleys (*n* = 10)	0	0 (0/13)	2.6 (2/78)
		Shopping baskets (*n* = 3)	0		
	Market 2	Shopping trolleys (*n* = 10)	1	0 (0/13)	
		Shopping baskets (*n* = 3)	0		
	Market 3	Shopping trolleys (*n* = 10)	1	15.4 (2/13)	
		Shopping baskets (*n* = 3)	1		
	Market 4	Shopping trolleys (*n* = 10)	0	0 (0/13)	
		Shopping baskets (*n* = 3)	0		
	Market 5	Shopping trolleys (*n* = 10)	0	0 (0/13)	
		Shopping baskets (*n* = 3)	0		
	Market 6	Shopping trolleys (*n* = 10)	0	0 (0/13)	
		Shopping baskets (*n* = 3)	0		
Heze (*n* = 40)	Market 7	Shopping trolleys (*n* = 10)	0	0 (0/13)	17.5 (7/40)
		Shopping baskets (*n* = 3)	0		
	Market 8	Shopping trolleys (*n* = 10)	3	23.1 (3/13)	
		Shopping baskets (*n* = 3)	0		
	Market 9	Shopping trolleys (*n* = 10)	4	28.6 (4/14)	
		Shopping baskets (*n* = 4)	0		
Zibo (*n* = 39)	Market 10	Shopping trolleys (*n* = 10)	0	0 (0/13)	2.6 (1/39)
		Shopping baskets (*n* = 3)	0		
	Market 11	Shopping trolleys (*n* = 10)	0	0 (0/13)	
		Shopping baskets (*n* = 3)	0		
	Market 12	Shopping trolleys (*n* = 10)	0	7.7 (1/13)	
		Shopping baskets (*n* = 3)	1		
Dezhou (*n* = 40)	Market 13	Shopping trolleys (*n* = 10)	0	14.3 (2/14)	7.5 (3/40)
		Shopping baskets (*n* = 4)	2		
	Market 14	Shopping trolleys (*n* = 10)	0	0 (0/13)	
		Shopping baskets (*n* = 3)	0		
	Market 15	Shopping trolleys (*n* = 10)	1	7.7 (1/13)	
		Shopping baskets (*n* = 3)	0		
Weifang (*n* = 29)	Market 16	Shopping trolleys (*n* = 10)	2	23.1 (3/13)	13.8 (4/29)
		Shopping baskets (*n* = 3)	1		
	Market 17	Shopping trolleys (*n* = 10)	1	7.7 (1/13)	
		Shopping baskets (*n* = 3)	0		
	Market 18	Shopping baskets (*n* = 3)	0	0 (0/3)	
Total (*n* = 226)	--	--	8.0 (18/226)	7.5 (17/226)	

**Table 3:** Antibiotic resistance rates for tested *S. aureus.*

Antimicrobials	No. of Resistant Isolates (%)					
	Animals (*n* = 11)	Raw Meats (*n* = 20)	Shopping Vehicles (*n* = 18)	MRSA (*n* = 20)	MSSA (*n* = 29)	Total (*n* = 49)
Ciprofloxacin	10 (90.9) ^a^	9 (45.0) ^b^	0 (0.0) ^c^	0 (0.0) ^a^	19 (65.5) ^b^	19 (38.8)
Tetracycline	8 (72.7) ^a^	11 (55.0) ^ab^	5 (27.8) ^bc^	5 (25.0) ^a^	19 (65.5) ^b^	24 (49.0)
Gentamicin	5 (45.5) ^a^	8 (40.0) ^a^	0 (0.0) ^b^	0 (0.0) ^a^	13 (44.8) ^b^	13 (26.5)
Kanamycin	6 (54.5) ^a^	9 (45.0) ^a^	16 (88.9) ^b^	16 (80.0) ^a^	15 (51.7) ^b^	31 (63.3)
Levofloxacin	2 (18.2) ^a^	3 (15.0) ^a^	0 (0.0) ^a^	0 (0.0) ^a^	5 (17.2) ^b^	5 (10.2)
Erythromycin	10 (90.9) ^ab^	16 (80.0) ^a^	18 (100.0) ^b^	19 (95.0) ^a^	25 (86.2) ^a^	44 (89.8)
Vancomycin	0 (0.0)	0 (0.0)	0 (0.0)	0 (0.0)	0 (0.0)	0 (0.0)
Penicillin	4 (36.4) ^a^	17 (85.0) ^b^	16 (88.9) ^b^	17 (85.0) ^a^	20 (69.0) ^a^	37 (75.5)
Linezolid	0 (0.0)	0 (0.0)	0 (0.0)	0 (0.0)	0 (0.0)	0 (0.0)
Clindamycin	8 (72.7) ^ab^	12 (60.0)^a^	17 (94.4)^b^	16 (80.0) ^a^	21 (72.4) ^a^	37 (75.5)
Florfenicol	9 (81.8) ^a^	6 (30.0) ^b^	0 (0.0) ^c^	0 (0.0) ^a^	15 (51.7) ^b^	15 (30.6)

The detection rates with different lowercase letters (^a^, ^b^ and ^c^) stand for significant differences (*p* < 0.05).

**Table 4:** Detection rates of resistance genes and virulence genes among isolates from different types of samples.

Genes	Number of Isolates Carrying Antibiotic Resistance and Virulence Genes (%)					
	Raw Meats (*n* = 20)	Shopping Vehicles (*n* = 18)	Animals (*n* = 11)	MRSA (*n* = 20)	MSSA (*n* = 29)	Total (*n* = 49)
Resistance						
*erm(A)*	0 (0.0)	0 (0.0)	0 (0.0)	0 (0.0)	0 (0.0)	0 (0.0)
*erm(B)*	5 (25.0) ^a^	12 (66.7) ^b^	5 (45.5) ^ab^	12 (60.0) ^a^	10 (34.5) ^a^	22 (44.9)
*erm(C)*	10 (50.0) ^a^	4 (22.2) ^a^	6 (54.5) ^a^	6 (30.0) ^a^	14 (48.3) ^a^	20 (40.8)
*vanA*	0 (0.0)	0 (0.0)	0 (0.0)	0 (0.0)	0 (0.0)	0 (0.0)
*vanB*	0 (0.0)	0 (0.0)	0 (0.0)	0 (0.0)	0 (0.0)	0 (0.0)
*cfr*	0 (0.0)	0 (0.0)	0 (0.0)	0 (0.0)	0 (0.0)	0 (0.0)
*poxtA*	0 (0.0)	0 (0.0)	0 (0.0)	0 (0.0)	0 (0.0)	0 (0.0)
*optrA*	0 (0.0)	0 (0.0)	0 (0.0)	0 (0.0)	0 (0.0)	0 (0.0)
Virulence						
*lukF-PV/lukS-PV*	0 (0.0) ^a^	3 (16.7) ^a^	0 (0.0) ^a^	3 (15.0) ^a^	0 (0.0) ^b^	3 (6.1)
*hla*	20 (100.0) ^a^	18 (100.0) ^a^	11 (100.0) ^a^	20 (100.0) ^a^	29 (100.0) ^a^	49 (100.0)
*hlb*	15 (75.0) ^a^	16 (88.9) ^a^	11 (100.0) ^a^	18 (90.0) ^a^	24 (82.8) ^a^	42 (85.7)
*eta*	14 (70.0) ^a^	6 (33.3) ^b^	3 (27.3) ^b^	6 (30.0) ^a^	17 (58.6) ^b^	23 (46.9)
*etb*	0 (0.0)	0 (0.0)	0 (0.0)	0 (0.0)	0 (0.0)	0 (0.0)
*fnbA*	0 (0.0)	0 (0.0)	0 (0.0)	0 (0.0)	0 (0.0)	0 (0.0)
*fnbB*	13 (65.0) ^ab^	9 (50.0) ^a^	10 (90.9) ^bc^	9 (45.0) ^a^	23 (79.3) ^b^	32 (65.3)
*sea*	6 (30.0) ^a^	1 (5.6) ^ab^	0 (0.0) ^bc^	4 (20.0) ^a^	3 (10.3) ^b^	7 (14.3)
*seb*	3 (15.0) ^a^	2 (11.1) ^a^	0 (0.0) ^a^	2 (10.0) ^a^	3 (10.3) ^a^	5 (10.2)
*sec*	0 (0.0)	0 (0.0)	0 (0.0)	0 (0.0)	0 (0.0)	0 (0.0)
*sed*	0 (0.0)	0 (0.0)	0 (0.0)	0 (0.0)	0 (0.0)	0 (0.0)
*see*	0 (0.0)	0 (0.0)	0 (0.0)	0 (0.0)	0 (0.0)	0 (0.0)
*tsst-1*	0 (0.0)	0 (0.0)	0 (0.0)	0 (0.0)	0 (0.0)	0 (0.0)

The detection rates with different lowercase letters (^a^, ^b^ and ^c^) stand for significant differences (*p* < 0.05).

**Table 5:** The virulence genes and resistance genes of whole-genome sequenced *S. aureus* isolates.

Strain	Source	MLST Types	Virulence Genes	Resistance Genes
2L5	Pig nasal swab in farm 1	398	exoenzyme genes (*aur*, *lip*, *sspA*, *sspB*, *sspC*), exotoxin genes (*hlgA*, *hlgB*, *hlgC*, *set17*, *set22*, *set24*, *set25*, *hla*, *hlb*, *hld*), effector delivery system genes (*esaA*, *essB*), immune modulation genes (*adsA*, *cap8D*, *cap8O*, *cap8L*, *cap8G*, *cap8P*, *cap8F*), nutritional/metabolic factor (*isdC*, *isdE*, *isdF*, *srtB*, *harA*), biofilm factor (*icaA*, *icaC*, *icaR*, *icaD*)	aminoglycoside resistance (*aac(6′)-aph(2″)*, *ant(6)-Ia*, *aadD*), fluoroquinolone resistance (*gyrA*: S84L, *grlA*: S80Y), tetracycline resistance (*tet(M)*, *tet(L)*), folate pathway antagonist resistance (*dfrG*), beta-lactam resistance (*bla*_Z_), amphenicol resistance (*fexA*), macrolide and lincosamide resistance (*erm(T)*, *erm(C)*), pleuromutilin and lincosamide resistance (*Isa(E)*), lincosamide resistance (*lnu(B)*)
HD15L3	Shopping trolley in supermarket 2	398	exoenzyme genes (*aur*, *lip*, *sspA*, *sspB*, *sspC*), exotoxin genes (*hlgA*, *hlgB*, *hlgC*, *set17*, *set22*, *set24*, *set25*, *hla*, *hlb*, *hld*), effector delivery system genes (*essA*, *essB*, *esaA*, *esaB*, *esaG*, *esxA)*, immune modulation genes (*adsA*, *capA*, *cap8B*, *cap8C*, *cap8D*, *cap8E*, *cap8F*, *cap8G*, *cap8M*, *capN*, *cap8O*, *cap8P*), nutritional/metabolic factor (*isdA*, *isdB*, *isdC*, *isdE*, *isdF*, *isdG*, *isdI*, *srtB*), biofilm factor (*icaA*, *icaC*, *icaR*, *icaD*)	aminoglycoside resistance (*aac(6′)-aph(2″)*, *ant(6)-Ia*, *aadD*), fluoroquinolone resistance (*gyrA*: S84L, *grlA*: S80Y), tetracycline resistance (*tet(M)*, *tet(L)*, *tet(K)*), folate pathway antagonist resistance (*dfrG*), beta-lactam resistance (*bla*_Z_), amphenicol resistance (*fexA*), macrolide and lincosamide resistance (*erm(T)*, *erm(C)*), pleuromutilin and lincosamide resistance (*Isa(E)*), lincosamide resistance (*lnu(B)*)
105L1	Raw meat in supermarket 19	6	adherence (*eap/map*), exoenzyme genes (*aur*, *splA*, *splB*, *splE*, *geh*, *sak*, *sspA*, *sspB*), exotoxin genes (*hlgA*, *hlgB*, *hlgC*, *lukD*, *lukE*, *sea*, *hla*, *hlb*, *hld*), effector delivery system genes (*essC*, *esaA*, *esaD*), immune modulation genes (*adsA*, *cap8D*, *cap8I*, *scn*), nutritional/metabolic factor (*isdA*, *isdB*, *isdC*, *isdE*, *isdF*, *srtB*, *harA*)	macrolide and lincosamide resistance (*erm(C)*), fluoroquinolone resistance (*grlA*: S80F), beta-lactam resistance (*bla*_Z_, *mecA*)
105L2	Raw meat in supermarket 19	6	adherence (*eap/map*), exoenzyme genes (*aur*, *splA*, *splB*, *splE*, *geh*, *sak*, *sspA*, *sspB*), exotoxin genes (*hlgA*, *hlgB*, *hlgC*, *lukD*, *lukE*, *sea*, *hla*, *hlb*, *hld*,), effector delivery system genes (*essC*, *esaA*, *esaD*), immune modulation genes (*adsA*, *scn*, *cap8D*, *cap8I*, *cap8K*, *cap8O*), nutritional/metabolic factor (*isdA*, *isdB*, *isdC*, *isdE*, *isdF*, *srtB*, *harA*), biofilm factor (*icaA*)	macrolide and lincosamide resistance (*erm(C)*), fluoroquinolone resistance (*grlA*: S80F), beta-lactam resistance (*bla*_Z_, two copies of *mecA*)
308	Raw meat in supermarket 20	6	adherence (*eap/map*), exoenzyme genes (*aur*, *splA*, *splB*, *splE*, *geh*, *sak*, *sspA*, *sspB*), exotoxin genes (*hlgA*, *hlgB*, *hlgC*, *lukD*, *lukE*, *sea*, *hla*, *hlb*, *hld*,), effector delivery system genes (*essC*, *esaA*, *esaD*), immune modulation genes (*adsA*, *scn*, *cap8D*, *cap8I*), nutritional/metabolic factor (*isdA*, *isdB*, *isdC*, *isdE*, *isdF*, *srtB*, *harA*)	macrolide and lincosamide resistance (*erm(C)*), fluoroquinolone resistance (*grlA*: S80F), beta-lactam resistance (*bla*_Z_, *mecA*)
HD33L3	Shopping trolley in supermarket 3	59	exoenzyme genes (*aur*, *lip*, *geh*, *sspA*, *sspB*, *sak*), exotoxin genes (*hlgA*, *hlgB*, *hlgC*, *set17*, *set18*, *set20*, *set22*, *set23*, *hla*, *hlb*, *hld*), effector delivery system genes (*esaA*, *essB*), immune modulation genes (*scn*, *cap8D*, *cap8I*, *cap8K*, *cap8L*, *cap8O*, *cap8P*), nutritional/metabolic factor (*isdA*, *isdB*, *isdC*, *isdE*, *isdF*, *srtB*), biofilm factor (*icaA*, *icaR*, *icaD*)	aminoglycoside resistance (*aph(3′)-III*, *ant(6)-Ia*), macrolide and lincosamide resistance (*erm(B)*), beta-lactam resistance (*mecA*)
HD36L3	Shopping basket in supermarket 3	338	exoenzyme genes (*aur*, *lip*, *geh*, *sspA*, *sspB*), exotoxin genes (*hlgA*, *hlgB*, *hlgC*, *set17*, *set18*, *set20*, *set22*, *set23*, *hla*, *hlb*, *hld*, *lukS-PV*, *seb*, *selq*, *selk*, *sek*, *seq*), effector delivery system genes (*esaA*, *essB*), immune modulation genes (*scn*, *cap8D*, *cap8I*, *cap8K*, *cap8L*, *cap8O*, *cap8P*), nutritional/metabolic factor (*isdA*, *isdB*, *isdC*, *isdE*, *isdF*, *srtB*), biofilm factor (*icaA*, *icaD*, *icaR*)	aminoglycoside resistance (*aph(3′)-III*, *ant(6)-Ia*), tetracycline resistance (*tet(K)*), macrolide and lincosamide resistance (*erm(B)*), beta-lactam resistance (*bla*_Z_, *mecA*)

## Data Availability

The genome sequences of the seven *S. aureus* isolates were deposited in the NCBI database and are publicly available under accession number PRJNA902522. Further inquiries can be directed to the corresponding authors.

## Supplementary Materials

The following supporting information can be downloaded at: https://www.mdpi.com/article/10.3390/foods15020248/s1, Table S1: Primer pairs used in this study [32,33,34,35,36,37,38,39,40]; Table S2: The isolates used for phylogenomic tree in this study.
