# Supplementary material for: Prevalence and Characterization of Methicillin-Resistant Staphylococcus aureus from Animals, Retail Meats and Market Shopping Vehicles in Shandong, China
Source: Foods. 2026 Jan 9;15(2):248. doi: 10.3390/foods15020248 (PMC12839714; doi:10.3390/foods15020248)
Supplement: Supplementary file 1 [file foods-15-00248-s001.zip › foods-3908642-supplementary.pdf]

Table S1. Primer pairs used in this study.

Gene	Primers	Sequences	Product size (bp)	annealing temperature (℃)	Reference
lukF-PV/*lukS*-PV	F	ATCATTAGGTAAAATGTCTGGACATGATCCA	433	58	[1]
	R	GCATCAASTGTATTGGATAGCAAAAGC			
sea	F	GGTTATCAATGTGCGGTGG	102	60	[2]
	R	CGGCACTTTTTTCTCTTCGG			
seb	F	GTATGGTGGTGTAACTGAGC	164	60	[2]
	R	CCAAATAGTGACGAGTTAGG			
sec	F	AGATGAAGTAGTTGATGTGTATGG	451	60	[2]
	R	CACACTTTAGAATCAACCG			
sed	F	CCAATAATAGGAGAAAATAAG	278	60	[2]
	R	ATTGGTATTTTTTTTOGTTC			
see	F	AGGTTTTTTCACAGGTCATCC	209	60	[2]
	R	CTTTTTTTTCTTCGTCAATC			
tsst-1	F	ACCCCTGTTCCCTTATCATC	326	57	[2]
	R	TTTTCAGTATTTGTAACGCC			
eta	F	GCAGGTGTTGATTTAGCATT	93	57	[2]
	R	AGATGTCCCTATTTTTGCTG			
etb	F	ACAAGCAAAAGAATACAGCG	226	57	[2]
	R	GTTTTTGGCTGCTTCTCTTG			
hla	F	AAAGGTACCATTGCTGGTC	604	58	[3]
	R	CAATTGGTAATCATCACGAAC			
hlb	F	TGTGGATTCGATAATGATAGC	482	61	[3]
	R	ACGTAGTAATATGGGAACGCT			
fnbA	F	GATACAAACCCAGGTGGTGG	191	56	[4]
	R	TGTGCTTGACCATGCTCTTC			
fnbB	F	TGTGCTTGACCATGCTCTTC	201	56	[4]
	R	AGTTGATGTCGCGCTGTATG			
cfr	F	TGAAGTATAAAGCAGGTTGGGAGTCA	746	57	[5]
	R	ACCATATAATTGACCACAAGCAGC			
erm(A)	F	TCTAAAAAGCATGTAAAAGAA	645	52	[6]
	R	CTTCGATAGTTTATTAATATTAG			
erm(B)	F	GAAAAGTACTCAACCAAATA	639	52	[6]
	R	AGTAACGGTACTTAAATTGTTTA			
erm(C)	F	TCAÅAACATAATATAGATAAA	642	52	[6]

	R	GCTAATATTGTTTAAATCGTCAAT			
optrA	F	TACTTGATGAACCTACTAACCA	422	57	[5]
	R	CCTTGAACTACTGATTCTCGG			
poxtA	F	GAACGCTTGGAGTATTTCGACTTC	778	57	[5]
	R	CTGGACTGAGAATACCCATC			
vanA	F	GGGAAAACGACAATTGC	732	59	[7]
	R	GTACAATGCGGCCGTTA			
vanB	F	ATGGGAAGCCGATAGTC	635	59	[7]
	R	GATTTCGTTCCTCGACC			
mecA	F	TGTCCGTAACCTGAATCAGC	519	57	[8]
	R	GACAAACTCCCACCTATCGC			
SCC*mec* I	F	GCTTTAAAGAGTGTCGTTACAGG	613	50	[9]
	R	GTTCTCTCATAGTATGACGTCC			
SCC*mec* II	F	CGTTGAAGATGATGAAGCG	398	50	[9]
	R	CGAAATCAATGGTTAATGGACC			
SCC*mec* III	F	CCATATTGTGTACGATGCG	280	50	[9]
	R	CCTTAGTTGTCGTAACAGATCG			
SCC*mec* IVa	F	GCCTTATTCGAAGAAACCG	776	50	[9]
	R	CTACTCTTCTGAAAAGCGTCG			
SCC*mec* IVb	F	TCTGGAATTACTTCAGCTGC	493	50	[9]
	R	AAACAATATTGCTCTCCCTC			
SCC*mec* IVc	F	ACAATATTTGTATTATCGGAGAGC	200	50	[9]
	R	TTGGTATGAGGTATTGCTGG			
SCC*mec* IVd	F	CTCAAAATACGGACCCCAATACA	881	50	[9]
	R	TGCTCCAGTAATTGCTAAAG			
SCC*mec* V	F	GAACATTGTTACTTAAATGAGCG	325	50	[9]
	R	TGAAAGTTGTACCCTTGACACC			

1. Lina, G.; Piemont, Y.; Godail-Gamot, F.; Bes, M.; Peter, M.O.; Gauduchon, V.; Vandenesch, F.; Etienne, J. Involvement of Panton-Valentine leukocidin-producing *Staphylococcus aureus* in primary skin infections and pneumonia. *Clin Infect Dis* **1999**, *29*, 1128-1132, doi:10.1086/313461.
2. Mehrotra, M.; Wang, G.; Johnson, W.M. Multiplex PCR for detection of genes for *Staphylococcus aureus* enterotoxins, exfoliative toxins, toxic shock syndrome toxin 1, and methicillin resistance. *J Clin Microbiol* **2000**, *38*, 1032-1035, doi:10.1128/JCM.38.3.1032-1035.2000.
3. Aarestrup, F.M.; Larsen, H.D.; Eriksen, N.H.; Elsberg, C.S.; Jensen, N.E. Frequency of alpha- and beta-haemolysin in *Staphylococcus aureus* of bovine and human origin. A comparison between pheno- and genotype and variation in phenotypic expression. *APMIS* **1999**, *107*, 425-430.
4. Arciola, C.R.; Campoccia, D.; Gamberini, S.; Baldassarri, L.; Montanaro, L. Prevalence of cna, fnbA and fnbB adhesin genes among *Staphylococcus aureus* isolates from orthopedic infections associated to different types of implant. *FEMS Microbiol Lett* **2005**, *246*, 81-86,

doi:10.1016/j.femsle.2005.03.035.
5. Brenciani, A.; Fioriti, S.; Morroni, G.; Cucco, L.; Morelli, A.; Pezzotti, G.; Paniccia, M.; Antonelli, A.; Magistrali, C.F.; Rossolini, G.M.; et al. Detection in Italy of a porcine *Enterococcus faecium* isolate carrying the novel phenicol-oxazolidinone-tetracycline resistance gene *poxtA*. *J Antimicrob Chemother* **2019**, *74*, 817-818, doi:10.1093/jac/dky505.
6. Silva, V.; Canica, M.; Ferreira, E.; Vieira-Pinto, M.; Saraiva, C.; Pereira, J.E.; Capelo, J.L.; Igrejas, G.; Poeta, P. Multidrug-Resistant Methicillin-Resistant Coagulase-Negative Staphylococci in Healthy Poultry Slaughtered for Human Consumption. *Antibiotics* **2022**, *11*, doi:10.3390/antibiotics11030365.
7. Moosavian, M.; Ghadri, H.; Samli, Z. Molecular detection of *vanA* and *vanB* genes among vancomycin-resistant enterococci in ICU-hospitalized patients in Ahvaz in southwest of Iran. *Infection and drug resistance* **2018**, *11*, 2269-2275, doi:10.2147/IDR.S177886.
8. Hososaka, Y.; Hanaki, H.; Endo, H.; Suzuki, Y.; Nagasawa, Z.; Otsuka, Y.; Nakae, T.; Sunakawa, K. Characterization of oxacillin-susceptible *mecA*-positive *Staphylococcus aureus*: a new type of MRSA. *J Infect Chemother* **2007**, *13*, 79-86, doi:10.1007/s10156-006-0502-7.
9. Zhang, K.; McClure, J.A.; Elsayed, S.; Louie, T.; Conly, J.M. Novel multiplex PCR assay for characterization and concomitant subtyping of *staphylococcal* cassette chromosome *mec* types I to V in methicillin-resistant *Staphylococcus aureus*. *J Clin Microbiol* **2005**, *43*, 5026-5033, doi:10.1128/JCM.43.10.5026-5033.2005.

Table S2. The isolates used for phylogenomic tree in this study

Genbank no.	strain	host	country	country label	MLST	MRSA
CP040625	JKD6004-DR	human	USA: Detroit, Michigan	U	ST239	Yes
AP025693.1	JICS127	human	Japan	J	ST8	Yes
CP012011	HC1340	human	Brazil: Rio de Janeiro city	B	ST239	Yes
CP012119	USA300_2014.C01	human	USA: Georgia	U	ST8	Yes
CP029667	AR_0225	human	USA	U	ST8	Yes
CP030137	M51	Animals	China	C	ST1516	Yes
CP031839	NX-T55	Animals	China: Ningxia	C	ST9	Yes
CP034102	O267	Animals	France	F	ST133	No
CP035003	PCFA-221	Animals	Korea	K	ST541	Yes
CP065199	DG36	Animals	Germany	G	ST9	Yes
CP094443	N10CSA27	human	China	C	unknown	Yes
CP022908	545	human	Germany	G	ST254	Yes
CP047801	UP_248	human	Germany	G	ST30	No
CP080560	HL17064	human	South Korea:Seoul	K	ST5	Yes
CP071594	PNID0137	human	South Korea: Busan	K	ST977	No
CP039448	VGC1	human	Taiwan: Taichung	C	ST59	Yes

CP040232	GD1706	human	China: Guangdong	C	ST4068	No
CP043386	NRS384	human	USA: Mississippi	U	ST8	Yes
CP045435	08-028	human	Brazil: Rio de Janeiro	B	ST1	Yes
CP047922	SR231	human	China:Hangzhou	C	ST965	Yes
CP053075	SA01	Food	China: Heilongjiang	C	ST6324	No
CP060141	2868B2	Food	China: Hangzhou	C	ST9	No
CP073012	SA14	human	China: Hubei	C	ST9	Yes
CP077098	RGB-095930	human	Germany: Regensburg, Bavaria	G	ST6610	Yes
CP077738	UMCG578	human	Netherlands: Groningen	N	ST398	Yes
CP077936	319	human	Germany: Augsburg	G	ST1708	No
CP082789	HL24830	human	South Korea	K	ST72	Yes
CP083259	HL25274	human	South Korea	K	ST45	Yes
CP087593	Newman NM-CQ	human	China: Chongqing	C	ST254	No
CP092556	VMRSA-WC062	human	USA	U	ST5	Yes
CP104020	831	human	USA: Birmingham, AL	U	ST8	Yes
LR027870	BPH2019	human	Australia	A	ST239	Yes
LR822060	P3.1	human	Argentina	Arg	ST5	Yes
AP017320	MI	human	USA:Michigan	U	ST5	Yes
AP019542	KG-03	human	Japan:Tokyo	J	ST5	Yes
AP020322	KUH180129	human	Japan: Kyoto	J	ST6313	No
AP024511	2007-13	human	Japan:Hokkaido	J	ST5	Yes
CP012692	FORC_027	human	South Korea: Seoul	K	ST5	Yes
CP016861	1969.N	human	USA: Ft. Benning, Georgia	U	ST8	Yes
CP017094	2148.C01	human	USA: Ft. Benning, Georgia	U	ST8	No
CP018766	UCI62	human	USA: California	U	ST5	Yes
CP029166	SVH7513	human	Australia: New South Wales	A	ST612	Yes
CP030138	M48	Animals	China	C	ST239	Yes
CP033987	P2D1C1	human	Israel: Jerusalem	ISR	ST5	Yes
CP094853	NY2010	human	China: Beijing	C	ST239	Yes
CP095119	IVB6154	Animals	Kenya:Marsabit	Ken	ST7619	No
CP100428	RMSA24	Food	China:Hefei	C	unknown	No
cp101123	MN8	human	USA	U	ST30	No

LR027873	BPH2070	human	Australia	A	ST239	Yes
AP019305	TUM9458	human	Japan	J	ST2389	No
CP012015	Gv51	human	Brazil: Teresina, PI	B	ST239	Yes
CP013231	UTSW MRSA 55	human	USA: Children Hospital, Dallas, Texas	U	ST8	Yes
CP015817	FORC_039	Food	South Korea	K	ST188	Yes
CP019590	C2406	human	Canada: Calgary	Ca	ST8	Yes
CP033112	ST20130944	human	France	F	ST30	No
CP042003	B3-14B	food	USA: Oklahoma	U	ST398	No
CP065515	GDY8P96A	Animals	China:Guangdong	C	ST1	No
JAPMTJ000000000	HD36L3	environment	China.Qingdao	C	ST338	Yes
JAPMTL000000000	HD15L3	environment	China.Qingdao	C	ST398	Yes
JAPMTK000000000	HD33L3	environment	China.Qingdao	C	ST59	Yes
JAPMTO000000000	105-1	food	China.Qingdao	C	ST6	Yes
JAPMTN000000000	105-2	food	China.Qingdao	C	ST6	Yes
JAPMTM000000000	308	food	China.Qingdao	C	ST6	Yes
JAPMTI000000000	2L5	Animals	China.Qingdao	C	ST398	No
CP017090	ISU935	Animals	USA	U	ST5	Yes
CP017804	ch21	Animals	"Poland: Wroclaw"	P	ST5	No
CP020019	08S00974	Animals	Germany	G	ST398	Yes
CP020467	CFSAN007896	environment	USA:IL	U	ST30	No
CP033977	P2D15C1	human	Israel: Jerusalem	Isr	ST5	Yes
CP094851	R20-14	human	China: Hangzhou	C	ST30	No
CP092538	VMRSA-WC123	human	USA	U	ST5	Yes
CP080562	HL21008	human	South Korea:Seoul	K	ST5	Yes
CP080249	NV_1	Food		C	ST5	Yes
CP077755	WHC09	Environment	China: WuHan	C	ST9	Yes
CP077741	NL1	human	Netherlands: Zwolle	N	ST398	Yes
CP039156	WCUH29	human	Poland	P	ST5	Yes
CP072116	TCH32929	human	USA	U	ST105	Yes
